# A Novel Defect Quantification Method Utilizing Multi-Sensor Magnetic Flux Leakage Signal Fusion

**DOI:** 10.3390/s24206623

**Published:** 2024-10-14

**Authors:** Wenlong Liu, Lemei Ren, Guansan Tian

**Affiliations:** School of Thermal Engineering, Shandong Jianzhu University, Jinan 250100, China; 19861806215@163.com (W.L.); tgs4170@sdjzu.edu.cn (G.T.)

**Keywords:** magnetic flux leakage (MFL), multi-sensor signal fusion (MSSF), characteristic approximation approach (CAA), defect depth calculation point

## Abstract

In the assessment of pipeline integrity using magnetic flux leakage (MFL) detection, it is crucial to quantify defects accurately and efficiently using MFL signals. However, in complex detection environments, traditional defect inversion methods exhibit low quantification accuracy and efficiency due to the complexity of their algorithms or excessive reliance on a priori knowledge and expert experience. To address these issues, this study presents a novel defect quantification method based on multi-sensor signal fusion (MSSF). The method employs a multi-sensor probe to fuse the MFL signals under multiple lift-off values, enhancing the diversity of defect information. This enables defect-opening profile recognition using the characteristic approximation approach (CAA). Subsequently, the MSSF method is based on a 3D magnetic dipole model and integrates the structural features of multi-sensor probes to develop an algorithm. This algorithm iteratively determines the defect depth at multiple data acquisition points within the defect region to obtain the maximum defect depth. The feasibility of the MSSF quantification method is validated through finite element simulation and physical experiments. The results demonstrate that the proposed method achieves accurate defect quantification while enhancing efficiency, with the number of iterations for each defect depth calculation point consistently requiring fewer than 15 iterations. For rectangular metal loss, perforation, and conical defects, quantification errors are less than 10%, meeting practical inspection requirements.

## 1. Introduction

As vital national strategic energy sources, oil and natural gas play a significant role in the national economy. Pipeline transportation serves as their primary mode of transportation. Currently, the combined length of oil and gas pipelines in different countries or regions has surpassed 2 million kilometers [1,2]. Due to the aging of pipelines and the harsh operating conditions they face, they are highly susceptible to various types of damage defects, such as metal loss, local pitting, cracks, dents, and corrosion, which increases the risk of oil and gas leakage [3]. Consequently, the integrity evaluation of pipeline structure is crucial for safe production and economic development [4,5,6]. As one of the most popular non-destructive testing technologies, magnetic flux leakage (MFL) detection technology is widely used in the detection of ferromagnetic materials like oil and gas pipelines [7,8], steel rails [9], steel wire ropes [10,11], steel belts [12], and other materials for automatic detection and evaluation.

Defect quantification is an essential part of pipeline MFL detection, with the most prevalent approach involving the construction of a robust model for determining defect size [13]. The key challenge lies in accurately and efficiently utilizing MFL signals to determine defect sizes, making it the focal point and primary difficulty in the defect quantification process [14]. In general, there are two main technical routes: (1) data-driven method and (2) model-driven method.

The data-driven method based on intelligent algorithms is constructed to describe the relationship between the signal and defect size. The majority of the method is based on machine-learning models. Initially, researchers could only quantify defects using shallow-learning methods due to limitations in computing capability. K. Hwang et al. [15] used a wavelet basis function (WBF) neural network to transform the MFL signal into a 3D defect profile, and the WBF approximated the defect profile by adjusting the resolution of the network. K.MR et al. [16] employed a neural network to extract the contour features of the MFL signal to identify the length and width dimensions of the defect and to estimate the depth of the defect using the peaks of the signal. B. Liu et al. [17] proposed a method for the reconstruction of 2D profiles based on a kernelized extreme learning machine (ELM) and used a quantum genetic algorithm (QGA) to optimize the parameters. However, shallow-learning methods can usually only obtain limited defect information due to the weak feature extraction capability [16]. In recent years, with the development of computer technology, deep learning methods have been widely applied to defect quantification. S.Lu et al. [18] proposed a new visual transform convolutional neural network (VT-CNN) for defect quantification, which uses a visual transform layer to transform the original MFL signal into a 3D image, improving quantification accuracy. Y.Ren et al. [19] designed a task-based bias DA network (TBDA-net) based on an adaptive dimension alignment subnet and task-based distribution-matching subnet to realize the quantification of defect size in small samples. M. Zhang et al. [20] proposed a visual deep transfer learning neural network to convert 1D MFL signals into 2D images, extending defect information and improving quantification accuracy. The above methods have made significant advances in defect quantification, but there are still some limiting factors: 1. Deep-learning models require a large number of labeled data for training, while in real industrial production, the limited availability of labeled samples and high production costs limit the performance of the models. 2. Deep-learning models are difficult to integrate with the underlying MFL theory, resulting in unclear physical meaning of the models.

The model-driven method leverages the uniqueness of the forward model, mapping defect size to defect MFL signals, to estimate defect size through iterative optimization of model parameters [21]. J. Feng et al. [22] considered the impact of detector vibration on the MFL detection in pipelines, and proposed a sensor lift-off modification method to correct the original MFL signal for accurate fault quantification. However, this correction process is time-consuming. G.Yu et al. [23] proposed a pipeline defect inversion method based on Stacking Learning, which enhances the generalization ability for different sample sets of defect inversion issues. Nevertheless, the quantification process requires the intervention of expert experience in the field of leakage detection, which reduces the quantification efficiency. R.Priewald et al. [24] used Gauss-Newton optimization to reconstruct the defect profile based on a nonlinear orthogonal model, but it is difficult to quantify the actual defects. Z. Wu et al. [25] proposed an algorithm based on an actor-critic structure for sizing defect profiles, aiming to mitigate the impact of signal noise on quantification and improve robustness. F.Li et al. [26] introduced a rapid approach to reconstruct defect profiles from MFL signals employing a modified harmony search (MHS) algorithm. Despite the increased computational speed, the quantification accuracy is compromised. D Zhang et al. [27] proposed a defect profile reconstruction method based on modified cuckoo search (MCS), employing a FEM as a forward model. The method successfully reconstructs 2D defect contours while enhancing computational speed by filtering and updating the primary error information between the predicted and reference signals, thereby minimizing interference from cluttered data in other dimensions. K.C. Hari et al. [28] proposed a novel scheme for the rapid forward simulation of MFL signals through a reduced nonlinear FEM method. This model facilitates the reconstruction of internal surface defect shapes using genetic algorithms (GAs), thereby improving the speed of defect quantification by decreasing the computational volume associated with FEM. S Zhang et al. [29] utilized an improved discrete magnetic dipole model (DMDM) as a forward model to estimate the depth of complex defects. Notably, the MFL signals obtained through the DMDM method exhibited minimal differences when compared with those derived from FEM and experimental results. Moreover, the computational efficiency of the DMDM approach was significantly greater than that of traditional FEM simulations. These methods provide theoretical support for defect quantification. Nevertheless, the complexity of the iterative inversion model results in low quantification efficiency, making their practical application in inspections challenging.

In addition to the two mainstream quantification methods, the data-driven method and model-driven method, recent research has explored the use of multiple sensor probes rather than the traditional single sensor. Scholars have investigated the use of specialized multi-sensor probe structures to develop defect size estimation equations. P.Shi et al. [30] analyzed the quantification errors associated with traditional single-sensor systems, particularly those arising from the lift-off effect and proposed a dual-sensor strategy that incorporates an uncertain lift-off value to enhance depth quantification accuracy. However, this method has not yet been validated through physical experiments. Y.Long et al. [31] introduced a detection topology for crack defects and, based on this, developed an MFL probe with double lift-off magnetic field sensors in the radial direction. This approach enhanced the quantification of crack defect depth and partially overcame the limitations of conventional magnetic flux leakage (MFL) detection methods in identifying and quantifying crack defects. Additionally, Y.Long et al. [32] proposed a new type of probe with dual diagonal distributed magnetic sensors. This design aims to compensate for probe attitude shifts caused by mechanical vibrations when the detector moves in the pipeline, thereby improving the signal-to-noise ratio and quantification accuracy. In conclusion, the use of multiple sensor probes enables the acquisition of MFL signals at varying lift-off values, facilitating lift-off compensation and enhancing defect quantification accuracy.

Based on the aforementioned analysis, this study proposes a novel defect quantification method based on multi-sensor signal fusion (MSSF), utilizing a configuration of four alternately staggered sensors. The multi-sensor probes enhance defect information diversity by integrating MFL signals from multiple lift-off values, significantly reducing the tedious iterative quantification process. This advancement markedly improves efficiency. Building on the fused signals, the MSSF method establishes a computational model for iteratively estimating defect depth, combining a three-dimensional magnetic dipole model (MDM) with the structural characteristics of the multi-sensor probe. Furthermore, this method identifies defect-opening profiles employing the characteristic approximation approach (CAA), leveraging MFL signals acquired by multi-sensor probes across various lift-off values. The proposed method represents a significant improvement in defect quantification efficiency and also improves the accuracy of MFL detection.

This paper is organized as follows: Section 2 elaborates on the defect quantification method based on the multi-sensor probe. Section 3 describes the finite element model (FEM) and physical experiments used to obtain the MFL signal of defects. Section 4 discusses and verifies the feasibility and robustness of the proposed method under simulation and experimental conditions. Finally, Section 5 presents the conclusions of this paper.

## 2. Methods

### 2.1. The Principle of the MFL Detection

**Figure 1:**
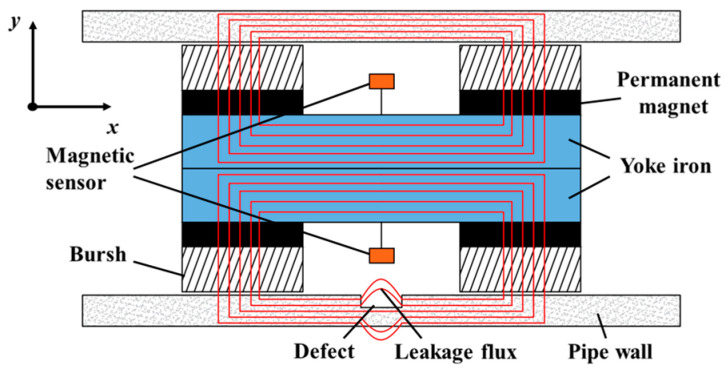
Principle of the MFL inspection.

Figure 1 illustrates the principle of internally detecting pipeline leakage magnetic field. The MFL detection model consists mainly of permanent magnets, magnetic sensors, and yoke irons. The principle is based on the high magnetic permeability characteristics of ferromagnetic materials. The pipe wall is fully magnetized using a permanent magnet and a rigid brush, achieving a saturated or near-saturated state. In the absence of any defects, the magnetic field lines run parallel to the inner surface of the pipe. However, the presence of defects, such as surface or near-surface imperfections, disrupts the magnetic field lines, leading to leakage from the pipe surface [33]. The characteristics of the leakage magnetic field distribution are closely correlated with the size of the defect. Detection and characterization of defects can be accomplished through the use of a magnetic sensor to detect the MFL signal.

### 2.2. The MSSF Method for Defect-Opening Profile Identification

**Figure 2:**
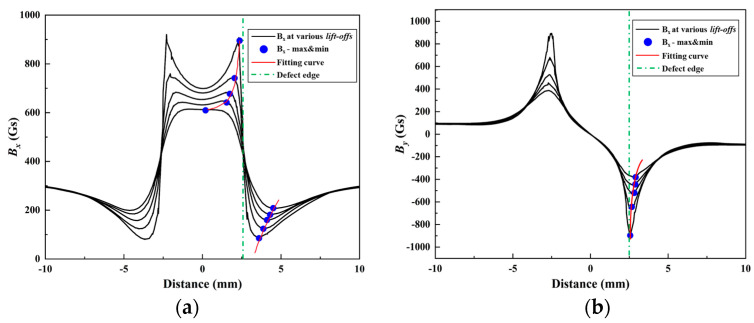
Defect-opening profile recognition: (**a**) *B_x_* in the *x*-detection, (**b**) *B_y_* in the *x*-detection.

Defect-opening profile identification is the initial step in defect quantification. This step significantly influences the accuracy of the quantification process. This section discusses the characteristic approximation approach (CAA) proposed by Yue Long for achieving accurate recognition of defect-opening profiles [34]. The CAA is based on the characteristic that the extreme value of the MFL signal approaches the edge of the defect as the lift-off value decreases. It calculates the signal pole value at the time when the lift-off value is 0 by numerically fitting the relationship between the pole value and lift-off value. Figure 2 shows that the extreme value point coordinates move closer to the defect edge (green dotted line) as *B_x_* and *B_y_* decrease with the lift-off value. Therefore, the CAA method is advantageous in identifying the defect profile compared to assuming that the peak width of the MFL signal is equal to the width of the defect. The CAA method determines the axial length of the defect using *B_x_*, *B_y_*, and *B_z_* in the axial detection direction. It identifies the circumferential length of the defect using *B_y_* and *B_z_* in the circumferential detection direction.

### 2.3. The MSSF Method for Defect Depth Quantification

In this section, the MSSF quantification method is accomplished by deriving a 3D MDM and utilizing a multi-sensor probe.

**Figure 3:**
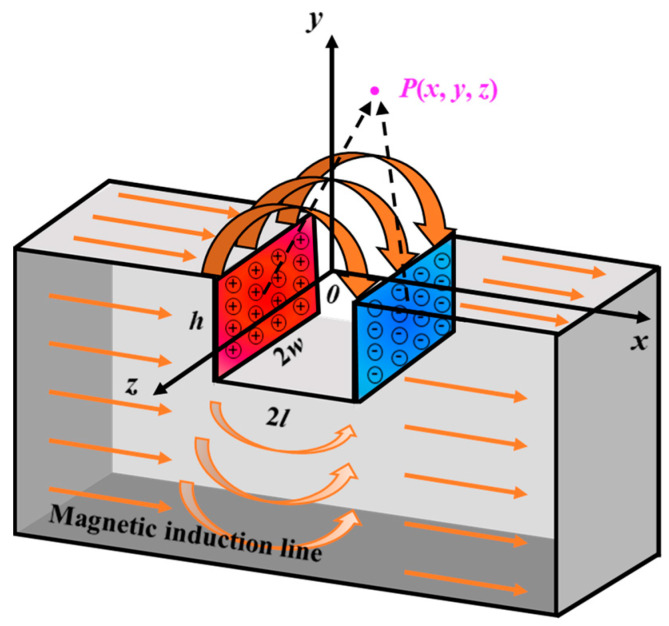
Three-dimensional MDM of the MFL detection.

Three-dimensional MDM: It is assumed that the two surfaces of a rectangular defect perpendicular to the applied magnetic field are uniformly distributed with magnetically opposite surface charges whose density is ±*σ_s_*. The interaction between the two produces a leakage magnetic field [35], as shown in Figure 3. A coordinate system with right angles is established on the inner surface of the pipe. The *x*-axis is defined as the axial direction of the pipe, the *y*-axis as the radial direction, and the *z*-axis as the circumferential direction. The dimensions of the defect are 2*l* for length, 2*w* for width, and *h* for depth. Equations (1)–(3) can be used to solve for the axial component *H_x_*, radial component *H_y_*, and circumferential component *H_z_* at the field point *P*(*x, y, z*) according to the Biot–Savart law [36,37].

(1) $$\begin{array}{ll} H_{x}\left(x,y,z\right)=\frac{\sigma_{s}}{4\pi }\times \{\arctan & \left[\frac{y\left(x+l\right)}{\left(z-w\right)\sqrt{(x+l{)}^{2}+(z-w{)}^{2}+y^{2}}}\right]-\arctan\left[\frac{y\left(x+l\right)}{\left(z+w\right)\sqrt{(x+l{)}^{2}+(z+w{)}^{2}+y^{2}}}\right]\\ & +\arctan\left[\frac{y\left(x-l\right)}{\left(z+w\right)\sqrt{(x-l{)}^{2}+(z+w{)}^{2}+y^{2}}}\right]-\arctan\left[\frac{y\left(x-l\right)}{[(z-w)\sqrt{(x-l{)}^{2}+(z-w{)}^{2}+y^{2}}}\right]\\ & +\arctan\left[\frac{\left(y+h\right)\left(x+l\right)}{\left(z+w\right)\sqrt{(x+l{)}^{2}+(z+w{)}^{2}+(y+h{)}^{2}}}\right]-\arctan\left[\frac{\left(y+h\right)\left(x-l\right)}{[(z+w)\sqrt{(x-l{)}^{2}+(z+w{)}^{2}+(y+h{)}^{2}}}\right]\\ & +\arctan\left[\frac{\left(y+h\right)\left(x-l\right)}{[(z-w)\sqrt{(x-l{)}^{2}+(z-w{)}^{2}+(y+h{)}^{2}}}\right]-\arctan\left[\frac{\left(y+h\right)\left(x+l\right)}{\left(z-w\right)\sqrt{(x+l{)}^{2}+(z-w{)}^{2}+(y+h{)}^{2}}}\right]\} \end{array}$$

(2) $$\begin{array}{ll} H_{y}\left(x,y,z\right)=\frac{\sigma_{s}}{4\pi }\times \{\ln & \left[\frac{\left[z+w+\sqrt{(x-l{)}^{2}+(z+w{)}^{2}+(y+h{)}^{2}}\right]\times \left[z+w+\sqrt{(x+l{)}^{2}+(z+w{)}^{2}+y^{2}}\right]}{\left[z-w+\sqrt{(x+l{)}^{2}+(z-w{)}^{2}+y^{2}}\right]\times \left[z+w+\sqrt{(x+l{)}^{2}+(z+w{)}^{2}+(y+h{)}^{2}}\right]}\right]\\ & +\ln\left[\frac{\left[z-w+\sqrt{(x-l{)}^{2}+(z-w{)}^{2}+y^{2}}]\times [z-w+\sqrt{(x+l{)}^{2}+(z-w{)}^{2}+(y+h{)}^{2}}\right]}{[z-w+\sqrt{(x-l{)}^{2}+(z-w{)}^{2}+(y+h{)}^{2}}\times [z+w+\sqrt{(x-l{)}^{2}+(z+w{)}^{2}+y^{2}}]}\right]\} \end{array}$$

(3) $$\begin{array}{ll} H_{z}\left(x,y,z\right)=\frac{\sigma_{s}}{4\pi }\times \{\ln & \left[\frac{\left[y+h+\sqrt{(x+l{)}^{2}+(z-w{)}^{2}+(y+h{)}^{2}}\right]\times \left[y+\sqrt{(x+l{)}^{2}+(z+w{)}^{2}+y^{2}}\right]}{\left[y+\sqrt{(x+l{)}^{2}+(z-w{)}^{2}+y^{2}}\right]\times \left[y+h+\sqrt{(x+l{)}^{2}+(z+w{)}^{2}+(y+h{)}^{2}}\right]}\right]\\ & +\ln\left[\frac{\left[y+\sqrt{(x-l{)}^{2}+(z-w{)}^{2}+y^{2}}]\times [y+h+\sqrt{(x-l{)}^{2}+(z+w{)}^{2}+(y+h{)}^{2}}\right]}{\left[y+h+\sqrt{(x-l{)}^{2}+(z-w{)}^{2}+(y+h{)}^{2}}]\times [y+\sqrt{(x-l{)}^{2}+(z+w{)}^{2}+y^{2}}\right]}\right]\} \end{array}$$

**Figure 4:**
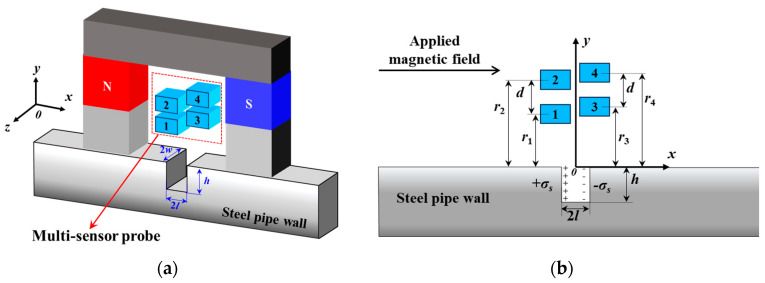
Multi−sensor probe detection system: (**a**) schematic of the spatial layout, (**b**) 2D dimensional display, blue squares represent sensors; 1, 2, 3, 4 represent sensor numbers.

Figure 4 shows the structure of the multi-sensor probe, which consists of four Hall sensors arranged in staggered rows above and below. The lift-off values are denoted as *r*_1_, *r*_2_, *r*_3_, and *r*_4_, respectively, where *r*_2_ − *r*_1_ = *d* and *r*_4_ − *r*_3_ = *d*. The CAA method can fit the MFL signals at a lift-off value of 0 from the MFL signals under the four different lift-off values. This enables the quantification of the defect length 2*l* and the defect width 2*w*.

**Figure 5:**
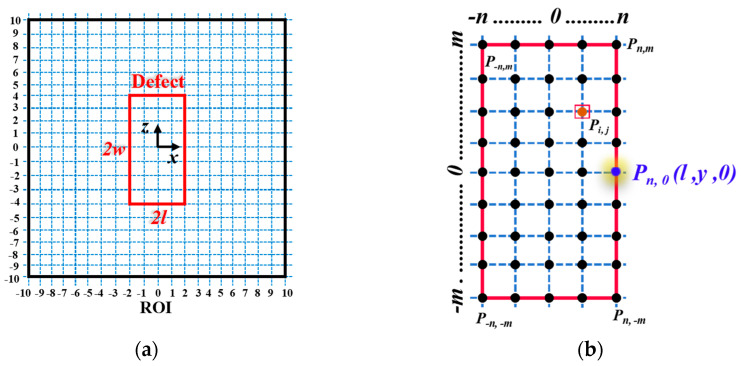
Detection region of the multi-sensor probe: (**a**) Region of interest (ROI) selected, (**b**) Defect-opening profile area.

When analyzing the MFL signals, particular attention should be paid to the signals in the vicinity of defects, as the magnetic field in regions far from the defect is very weak and exhibits negligible differences [29]. In this study, a square signal area with a side length of 20 mm is chosen as an example, with its center coinciding with the defect center. This area adequately covers the leakage magnetic field generated by the defect. As shown in Figure 5a, the Region of Interest (ROI) is delineated by blue dashed lines, representing detection channels along the *x* and *z* directions, spaced 1 mm apart. The defect-opening profile identified by CAA is depicted by the red box in Figure 5b, encompassing 2*m* + 1 channels in the *x* direction and 2*n* + 1 channels in the *z* direction. There are *c* = (2*m* + 1) × (2*n* + 1) data acquisition points, *P_−n, −m_, P_−n, m_, P_n, −m_, P_n, m_,* ⋯⋯*P_i,j_*, *i*∈[−n, n], *j*∈[−m, m]. The magnetic field at point *P_i,j_* ($\frac{i}{n}l,\;y,\;\frac{j}{m}w$) is given by

(4) $$H_{P\;ij}(\frac{i}{n}l,\;y,\;\frac{j}{m}w)=(H_{\;x}(\frac{i}{n}l,\;y,\;\frac{j}{m}w),\;H_{\;y}(\frac{i}{n}l,\;y,\;\frac{j}{m}w),\;H_{\;z}(\frac{i}{n}l,\;y,\;\frac{j}{m}w))$$

where *y* represents the lift-off value of data acquisition points, *y* > 0. By calculating the defect depth at each data acquisition point, the maximum defect depth can be determined using Equation (5), providing insights into the extent of pipeline damage.

(5) $$h_{max}={max\left\{h_{1,1},{\;h}_{1,2}\cdots \cdots h_{i,j}\right\}}_{-n<i<n,\;-m<j<m}$$

Taking point *P_n,0_* (*l,y,0*) at the center of the defect edge in Figure 5b as an example, we derive the algorithm for solving defect depth using the MSSF based on the 3D MDM. By substituting point *P_n,0_* (*l,y,0*) into Equations (1)–(3), expressions for *H_x_*, *H_y_*, and *H_z_* at this point are obtained as (6)–(8).

(6) $$H_{x}\left(l,y,0\right)=\frac{\sigma_{s}}{4\pi }\times \left\{\arctan\left[\frac{4l\left(y+h\right)}{w\sqrt{{4l}^{2}+w^{2}+(y+h{)}^{2}}}\right]-\arctan\left[\frac{4ly}{w\sqrt{{4l}^{2}+w^{2}+y^{2}}}\right]\right\}$$

(7) $$H_{y}\left(l,y,0\right)=\frac{\sigma_{s}}{4\pi }\times \left\{\ln\left[\frac{\left[w+\sqrt{w^{2}+(y+h{)}^{2}}\right]\times \left[w+\sqrt{4l^{2}+w^{2}+y^{2}}\right]\times [\sqrt{w^{2}+y^{2}}-w]\times [\sqrt{4l^{2}+w^{2}+(y+h{)}^{2}}-w]}{\left[\sqrt{4l^{2}+w^{2}+y^{2}}-w\right]\times \left[w+\sqrt{4l^{2}+w^{2}+(y+h{)}^{2}}\right]\times [\sqrt{w^{2}+(y+h{)}^{2}}-w]\times [w+\sqrt{w^{2}+y^{2}}]}\right]\right\}$$

(8) $$H_{z}\left(l,y,0\right)=\frac{\sigma_{s}}{4\pi }\times \left\{\ln\left[\frac{\left[y+h+\sqrt{4l^{2}+w^{2}+(y+h{)}^{2}}\right]\times \left[y+\sqrt{4l^{2}+w^{2}+y^{2}}\right]}{\left[y+\sqrt{4l^{2}+w^{2}+y^{2}}\right]\times \left[y+h+\sqrt{4l^{2}+w^{2}+(y+h{)}^{2}}\right]}\right]\right\}=0$$

During the derivation of the formula, it was found that the signal value of the circumferential signal *H_z_* at the point *P_n,0_* (*l*, *y*, *0*) is zero. This is because the circumferential signal is insensitive to defect edges perpendicular to the direction of magnetization. However, it is more sensitive to defect edges parallel to the direction of magnetization and to defect right-angled features [38]. Thus, the significance of the circumferential signal *H_z_* at this point is not relevant. Therefore, subsequent calculations mainly utilize the axial signal *H_x_* and radial signal *H_y_*. The intermediate variables $P_{x}\left(y\right),$ $M_{y}(y)$, and $N_{Z}\left(y\right)\;$are defined as follows:

(9) $$P_{x}(y)=tan(H_{x}\times \frac{4\pi }{\sigma_{s}})$$

(10) $$M_{y}(y)=e^{H_{y}\times \frac{4\pi }{\sigma_{s}}}$$

(11) $$N_{z}(y)=e^{H_{z}\times \frac{4\pi }{\sigma_{s}}}\;=\;1$$

In Equations (9) and (10), we observe that the numerator and denominator of $P_{x}(y)$ and $M_{y}(y)$ are of the same order. Inspired by [32], we divide the numerator and denominator of $P_{x}(y)$ by *y^2^* and the numerator and denominator of $M_{y}(y)$ by *y*^4^. Additionally, we define the intermediate variables *u*(*y*), *v*(*y*), and *η*(*y*).

(12) $$u\left(y\right)=l/y$$

(13) $$v\left(y\right)=w/y$$

(14) $$\eta \left(y\right)=h/y$$

Therefore, Equations (9) and (10) can be expressed as (15) and (16), respectively.

(15) $$P_{x}(y)=\frac{4u(y)(1+\eta (y))}{v(y){\left(4{u(y)}^{2}+{v(y)}^{2}+1+2\eta (y)+{\eta (y)}^{2}\right)}^{\frac{1}{2}}}-\frac{4u(y)}{v(y){\left(4{u(y)}^{2}+{v(y)}^{2}+1\right)}^{\frac{1}{2}}}$$

(16) $$\begin{array}{cc} M_{y}(y) & =\frac{v(y)+{\left(1+2\eta (y)+{\eta (y)}^{2}+{v(y)}^{2}\right)}^{\frac{1}{2}}}{{\left(4{u(y)}^{2}+{v(y)}^{2}+1\right)}^{\frac{1}{2}}-v(y)}\times \frac{v(y)+{\left(4{u(y)}^{2}+{v(y)}^{2}+1\right)}^{\frac{1}{2}}}{v(y)+{\left(4{u(y)}^{2}+{v(y)}^{2}+1+2\eta (y)+{\eta (y)}^{2}\right)}^{\frac{1}{2}}}\\ & \times \frac{{\left({v(y)}^{2}+1\right)}^{\frac{1}{2}}-v(y)}{{\left({v(y)}^{2}+1+2\eta (y)+{\eta (y)}^{2}\right)}^{\frac{1}{2}}-v(y)}\times \frac{{\left(4{u(y)}^{2}+{v(y)}^{2}+1+2\eta (y)+{\eta (y)}^{2}\right)}^{\frac{1}{2}}-v(y)}{v(y)+{\left({v(y)}^{2}+1\right)}^{\frac{1}{2}}} \end{array}$$

The Equations (15) and (16) can be solved iteratively to determine the value of *η*(*y*). For sensors 1, 2, 3, and 4, the relation $r_{2}=r_{1}+d$ and $r_{4}=r_{3}+d$ holds. Therefore, *h* can be denoted by $\eta \left(r_{1}\right)$, $\eta \left(r_{2}\right)$, $\eta \left(r_{3}\right)$, $\eta \left(r_{4}\right)$, and *d*, thereby enabling multi-sensor signal fusion to quantify the defects. Please refer to Equations (17) and (18) for further details:

(17) $$\left\{\begin{array}{c} h_{1}=\frac{\eta (r_{1})\eta (r_{2})d}{\eta (r_{1})-\eta (r_{2})}\\ h_{2}=\frac{\eta (r_{3})\eta (r_{4})d}{\eta (r_{3})-\eta (r_{4})} \end{array}\right.$$

(18) $$h=\alpha h_{1}+\beta h_{2}$$

where α and β are the weighting coefficients for $h_{1}$ and $h_{2}$, weighted average solution *h*.

**Figure 6:**
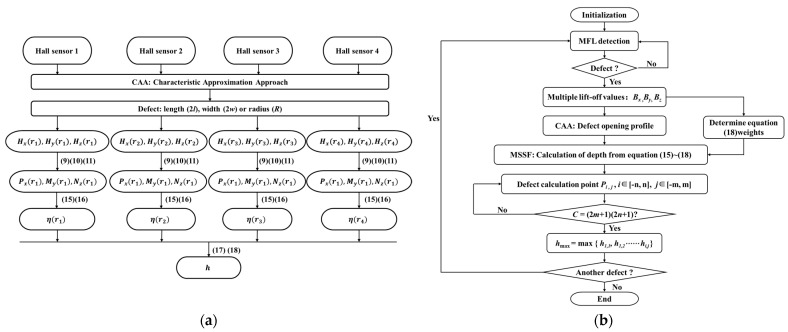
The MSSF quantification algorithm: (**a**) the solution map, (**b**) the block diagram.

The solution map and block diagram of the MSSF quantification are shown in Figure 6a,b respectively. In the solution map, for sensor 1 with a lift-off value of $r_{1}$, the obtained values of *H_x_* ($r_{1}$), *H_y_* ($r_{1}$), *H_z_* ($r_{1}$) and the dimensions of the defect length 2*l* and width 2*w* are substituted into Equations (9), (10), and (11) to solve for $P_{x}(y),M_{y}(y)$, and $N_{Z}(y)$. Subsequently, iterative computation of Equations (15) and (16) are used to obtain $\eta \left(r_{1}\right)$. Similarly, $\eta \left(r_{2}\right)$, $\eta \left(r_{3}\right)$, and $\eta \left(r_{4}\right)$ are obtained for sensors 2, 3, and 4. Finally, Equations (17) and (18) are used to estimate the defect depth *h*.

The block diagram illustrates the complete procedure for defect quantification, as follows:

Step 1: Conduct an MFL detection on the component to detect the presence of defects. If a defect is identified, the region surrounding the defect is designated as the ROI, and Step 2 is initiated. If no defects are found, the process is completed.

Step 2: Use multi-sensor probes to extract MFL signals at various lift-off values. Apply CAA to identify defect-opening profiles. These profiles are then substituted into Equations (15)–(18) to iteratively determine the depth of each point *P_i,j_* within the computational domain.

Step 3: After computing the depth *h* at all calculation points within the domain, where *c* = (2*m+*1) × (2*n+*1), output the maximum depth *h_max_* from all calculated values. If the depth calculations for all points are not yet completed, proceed to compute the depth for the next point.

Step 4: Check for additional defects. If no further defects are detected, conclude the quantification process. If other defects are present, continue with the quantification process for the next defect.

For defect depths at other data acquisition points, the derivation process is identical to the above, allowing computation of defect depths at all sampling points within the defect-opening region to ultimately obtain the maximum depth *h_max_*. Similarly, for cylindrical [35] and conical [39] defects, the MSSF quantification method can be utilized for depth calculation. The derivation process parallels that of rectangular defects, with the defect-opening dimensions 2*l* and 2*w* replaced by the radius *R*. The specific transformations are as follows:

Defect-opening dimensions: length 2*l,* width 2*w* → radius *R*

$$\mathrm{Data}\;\mathrm{sampling}\;\mathrm{points}:\;P_{i,j}\;(\frac{i}{n}l,\;y,\;\frac{j}{m}w)\;\to \;P_{i,j}\;(\frac{i}{n}R,\;y,\;\frac{j}{m}R)$$

$$\mathrm{Intermediate}\;\mathrm{variables}:\;\left\{\begin{array}{c} u\left(y\right)=l/y\\ v\left(y\right)=w/y\\ \eta \left(y\right)=h/y \end{array}\right.\;\to \;\left\{\begin{array}{c} u\left(y\right)=R/y\\ \eta \left(y\right)=h/y \end{array}\right.$$

## 3. Simulation and Experiment

### 3.1. Design of the Finite Element Model

**Figure 7:**
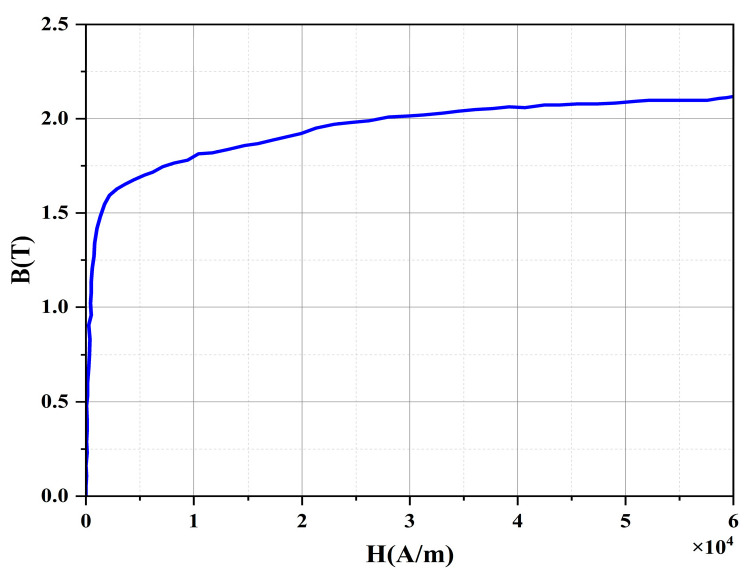
Magnetostatic characteristics of nonlinear *B-H* curves.

FEM is based on Maxwell equations when solving MFL fields. MFL problems can be effectively treated as magnetostatic problems using a scalar magnetic potential method. These magnetostatic problems can be expressed by the following equation [40]:

(19) $$\nabla \times \frac{1}{\mu }\;\nabla \times \;A\;=\;J$$

where ∇ is the Hami operator, *A* is the magnetic vector potential, *J* is the current density, and *μ* is a function of the magnetic flux density *B*, given by *μ* = *μ*(*B*), which exhibits nonlinearity as depicted in Figure 7, corresponding to the material’s *B-H* curve.

The integration by parts in (19) gives the following [41]:

(20) $$∭\frac{1}{\mu }\left(\frac{\partial W}{\partial x}\times \frac{\partial A}{\partial x}+\frac{\partial W}{\partial y}\times \frac{\partial A}{\partial y}+\frac{\partial W}{\partial z}\times \frac{\partial A}{\partial z}\right)da-∭w\times J\;da=0$$

where *w* and *a* denote the weight function and volume of the space, respectively. As the shape functions are equal to the weighting functions [42], Equation (20) could be represented in a matrix from Equation (21):

(21) $$\sum_{e}\left[\frac{1}{\mu }[S{]}_{e}A_{e}-\left[Q{]}_{e}J\right]=[0\right]$$

(22) $$S_{e,ij}=∭\frac{\partial N_{i}}{\partial x}\frac{\partial N_{j}}{\partial x}+\frac{\partial N_{i}}{\partial y}\frac{\partial N_{j}}{\partial y}+\frac{\partial N_{i}}{\partial z}\frac{\partial N_{j}}{\partial z}da$$

(23) $$Q_{e,ij}=∭N_{i}da$$

where *e* represents that the matrices pertain to a specific element, and *S_e,ij_*, and *Q_e,ij_* are the typical entries in these matrices in (21). After calculating the magnetic vector potential *A_e_* at each vertex of the element in the magnetic field from (21), then the magnetic field strength *B* can be calculated from *B* = ∇ × *A*.

**Figure 8:**
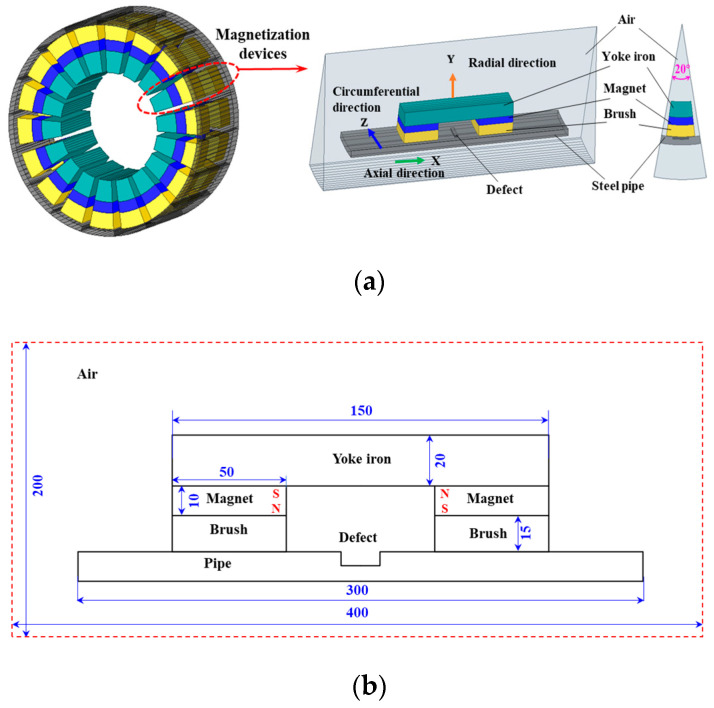

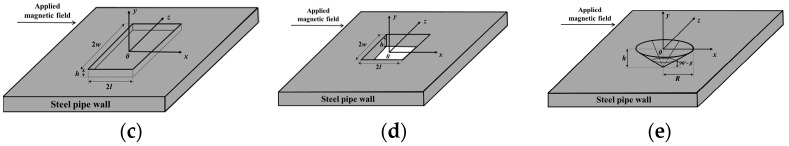
The FEM of MFL detection: (**a**) MFL detection system, (**b**) structural dimensions of the MFL detection system, (**c**) rectangular metal loss defect, (**d**) square perforation defect, (**e**) conical defect.

To accurately obtain the distribution characteristics of the leakage magnetic field of defects and verify the feasibility of the MSSF quantification method, the research team established a 3D FEM using a ANSYS 2021 software, as shown in Figure 8a. To reduce computational complexity, only 1/18th of the entire circumference is selected for the simulation study, due to the cylindrical symmetry of both the leakage detector and the inspected pipeline. The ferromagnetic specimen in the model is made of Q235 steel. Its magnetic permeability adheres to the *B-H* curve illustrated in Figure 6. The magnetization device comprises a yoke and two permanent magnets made of neodymium with a remanent magnetic strength of 1.3 T. A layer of rigid brushes is placed on the surface of the permanent magnets, which are in direct contact with the ferromagnetic specimen. The entire MFL detection device is enclosed by the air domain, where the relative magnetic permeability is set to 1. The detailed structural and material parameters of the detector are illustrated in Figure 8b and Table 1, respectively.

Given the variability in actual pipe defect profiles, this section analyzes the MFL signals of rectangular defect, square perforation defect, and conical defect using FEM, as illustrated in Figure 8c–e. The model does not account for velocity effects, so the leakage magnetic field can be treated as static. Therefore, the 3D static magnetic field solver is used. To resolve the contradiction between the large specimen volume and the same defect volume, we implemented varying grid sizes in different regions [43]. The computational domain was divided into unstructured grids, and the air domain over the defects was locally encrypted.

### 3.2. Results of the FEM Simulation

**Figure 9:**
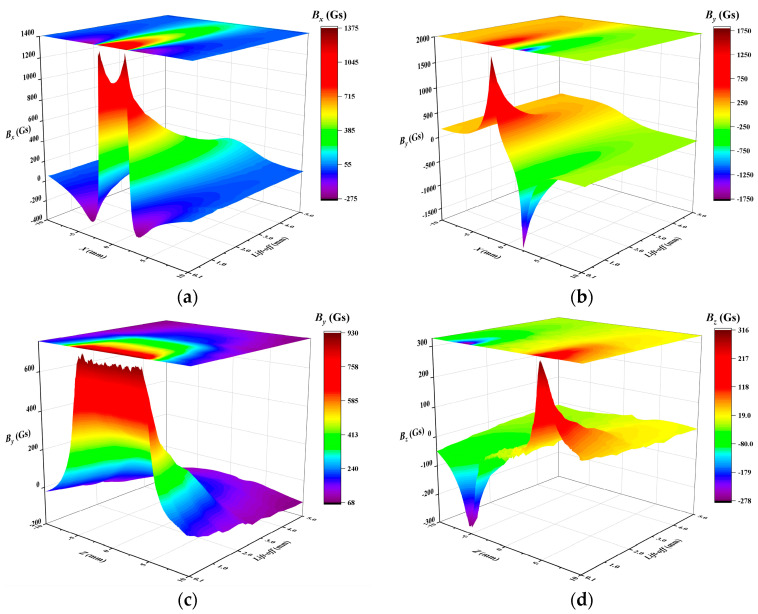
The correlation between rectangular metal loss defect leakage magnetic field and the lift-off value: (**a**) *B_x_* in the *x*-detection, (**b**) *B_y_* in the *x*-detection, (**c**) *B_y_* in the *z*-detection, (**d**) *B_z_* in the *z*-detection.

The rectangular metal loss defect dimensions chosen for this study are length 2*l* = 4 mm, width 2*w* = 10 mm, depth *h* = 3 mm, and wall thickness *t* = 10 mm. MFL signals were simulated across lift-off values ranging from 0.1 mm to 5 mm, incremented by 0.1 mm. Figure 9 shows the relationship between the leakage magnetic field and the lift-off value in the *x* and *z* detection directions, as simulated by the FEM. It is evident that as the lift-off value increases, the amplitude of each signal component decreases sharply, causing the characteristic information, such as extreme and inflection points, to become gradually submerged, and the signal-to-noise ratio (SNR) to decrease. *B_x_* in the *x*-detection direction and *B_y_* in the *z*-detection direction show axisymmetric single-peak features, while *B_y_* in the *x*-detection direction and *B_z_* in the *z*-detection direction show centrosymmetric double-peak features. The signal components in the *x*-detection direction have a peak spacing that corresponds to the length of the defect 2*l*. Similarly, the signal components in the *z*-detection direction have a peak spacing that corresponds to the width of the defect 2*w*. This is a prerequisite for implementing the CAA method.

**Figure 10:**
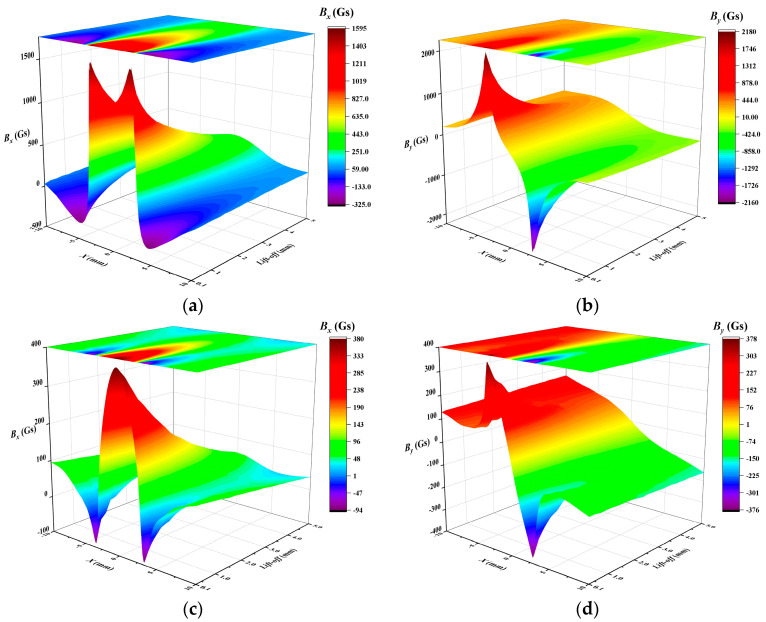
The correlation between the leakage magnetic field in the *x*-detection and the lift-off value: (**a**) *B_x_* for square perforation defect, (**b**) *B_y_* for square perforation defect, (**c**) *B_x_* for conical defect, (**d**) *B_y_* for conical defect.

Similarly, the square perforation defect measures 2*l* = 6 mm and 2*w* = 6 mm, with a defect depth *h* = wall thickness *t* = 3 mm. The conical defect has dimensions *R* = 3 mm, *h* = 3 mm, *β* = 45°, and wall thickness *t* = 10 mm. MFL signals are simulated across a range of lift-off values from 0.1 mm to 5 mm. Figure 10 illustrates the axial signal *B_x_* and radial signal *B_y_* in the *x*-detection direction for both of these defects.

### 3.3. Design of the Physical Experiments

**Figure 11:**
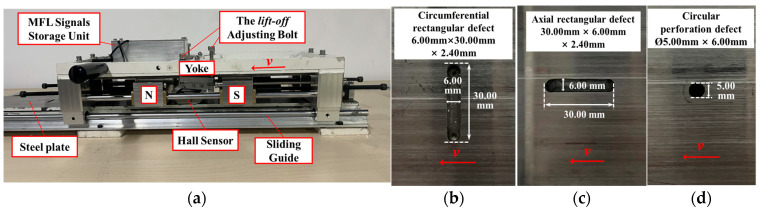
Experiment site: (**a**) MFL detection system, (**b**) circumferential rectangular groove defect, (**c**) axial rectangular groove defect, (**d**) circular perforation defect.

To evaluate the practicality of the MSSF quantification method in practical inspection, the research team developed an MFL detection device and a ferromagnetic specimen with surface defects, as shown in Figure 11. The steel plate specimen measures 440 mm in length and has a wall thickness *t* of 6.00 mm. It contains a circumferential rectangular groove defect with an axial length 2*l* of 6.00 mm, a circumferential width 2*w* of 30.00 mm, and a depth *h* of 2.40 mm, an axial rectangular groove defect with an axial length 2*l* of 30.00 mm, a circumferential width 2*w* of 6.00 mm, and a depth *h* of 2.40 mm, as well as a circular perforation defect with a radius *R* of 2.50 mm and a depth *h* of 6.00mm. The specimen was magnetized by a permanent magnet with a remanent magnetic strength of 1.3 T.

The MFL signal collection probe consists of four Hall sensors. These sensors maintain an axial sampling interval of 0.05 mm, while the detection device moves at a speed of 0.1 m/s along the slide guide. The sensor lift-off value is adjusted using a fixing bolt. Upon completion of detection, the collected MFL signal is stored in the storage unit and subsequently transmitted to the data analysis and processing system.

### 3.4. Results of the Physical Experiments

**Figure 12:**
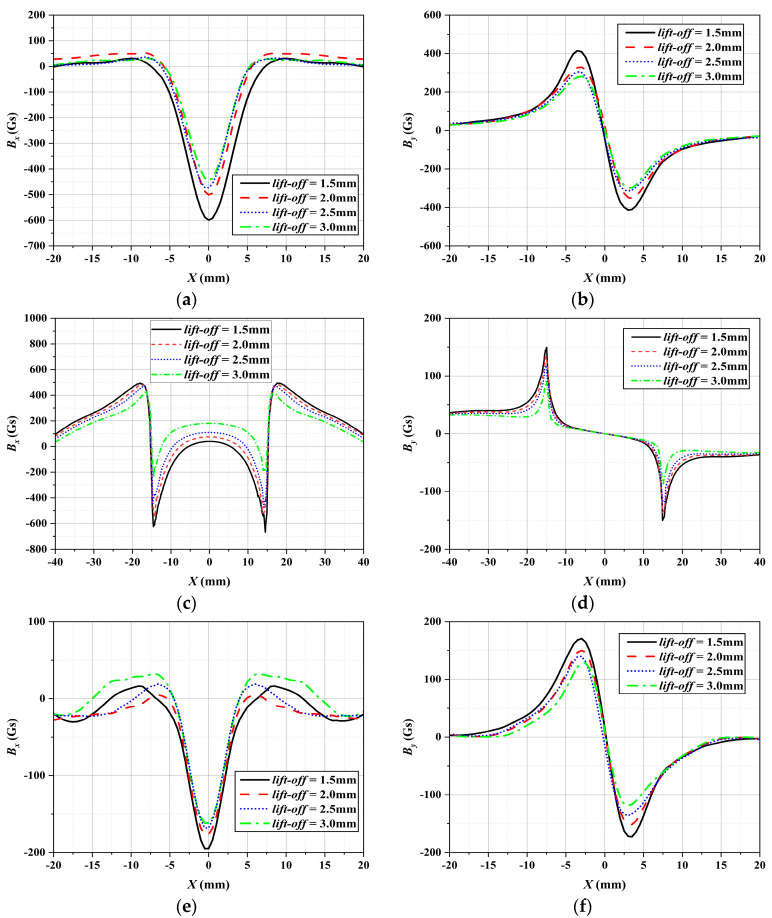
The MFL signals obtained from the experiments: (**a**) *B_x_* of the circumferential rectangular groove defect, (**b**) *B_y_* of the circumferential rectangular groove defect, (**c**) *B_x_* of the axial rectangular groove defect, (**d**) *B_y_* of the axial rectangular groove defect, (**e**) *B_x_* of the circular perforation defect, (**f**) *B_y_* of the circular perforation defect.

During the experiment, the adjusting bolts were used to set four different lift-off values: *r*_1_ = 1.5 mm, *r*_2_ = 2.5 mm, *r*_3_ = 2.0 mm, and *r*_4_ = 3.0 mm, with a sensor spacing *d* of 1 mm. Several experiments were conducted, and the results are presented in Figure 12, illustrating the measured MFL signals of rectangular grooves and perforation defects in the *x*-direction detection. It becomes evident that as the lift-off value increases, the MFL signal exhibits a consistent trend with the FEM results, demonstrating a decrease in signal amplitude and SNR. It is noteworthy that the amplitude of the MFL signal obtained experimentally may not match exactly with that obtained by the FEM due to differences in material properties and dimensional setup parameters. However, the signals’ characteristics and distribution trends exhibit a high degree of consistency.

## 4. Results and Discussion

In this section, the functions and robustness of the MSSF quantification method will be discussed based on the simulation data obtained from the FEM and the experimental data collected from the MFL detection experiment.

### 4.1. The Solution of the Size of Rectangular Defect

Based on the rectangular metal loss defect MFL signals presented in Figure 9, the lift-off values for the multi-sensor probe are selected as follows: *r*_1_ = 1 mm, *r*_2_ = 2 mm, *r*_3_ = 1.5 mm, *r*_4_ = 2.5 mm, with a sensor spacing *d* of 1 mm. The CAA method was used to estimate the length and width dimensions of defects based on the MFL signals under the different lift-off values. Within the defect-opening region, there are 11 channels in the *x*-detection direction and 5 channels in the *y*-detection direction, and the total number of data acquisition points *c* is 55. The defect depth *h* at each data acquisition point was estimated using *H_x_*(*r*), *H_y_*(*r*), *H_z_*(*r*), and *d*, following the algorithm outlined in Figure 6. The specific calculation results are shown in Table 2.

Similarly, the dimensions of the circumferential and axial rectangular groove defects were estimated using the MSSF quantification method, based on the experimental data shown in Figure 12a–d. The specific calculation results are shown in Table 3 and Table 4.

Table 2, Table 3 and Table 4 demonstrate that the error between the estimated and actual defect size derived from the MSSF quantification method is below 10% for both simulated and experimental cases. Notably, the axial and circumferential sampling intervals of the sensors were notably larger in the physical experiments compared to the FEM simulation. Consequently, this reduces the accuracy of defect-opening profile recognition by CAA, which in turn increases the depth quantification error. However, the error remains within acceptable thresholds. Regarding computation time, due to larger defect sizes observed in experiments compared to simulations, there is an increase in data acquisition points, resulting in longer computing times for quantifying experimental defects.

### 4.2. The Solution of the Size of Perforation Defect

To investigate the proposed method’s versatility in dealing with various types of defects, this study also examined perforation defects through quantitative analysis. The axial signal *B_x_* and radial signal *B*_y_ in the *x*-detection direction are illustrated in Figure 10a,b, respectively, for the simulated signals of the square perforation defect. The lift-off values for the multi-sensor probe were selected as follows: *r*_1_ = 1 mm, *r*_2_ = 2 mm, *r*_3_ = 1.5 mm, and *r*_4_ = 2.5 mm. The specific calculation results are shown in Table 5. Table 5 quantification results and relative errors for the square perforation defect using simulation data

The experiment estimated the defect size utilizing the MSSF quantification method that relied on the MFL signals of the circular perforation defect shown in Figure 12e,f. The calculation results are presented in Table 6.

Table 5 and Table 6 demonstrate the high accuracy and efficiency of the MSSF quantification method in handling various shapes of perforation defects. The size quantification errors for the square perforation defect in the FEM simulation are below 5%. In the physical experiment, the size quantification errors for the circular perforation defect are below 10%, and the calculation time of both is less than 5 s.

### 4.3. The Solution of the Size of Conical Defect

**Figure 13:**
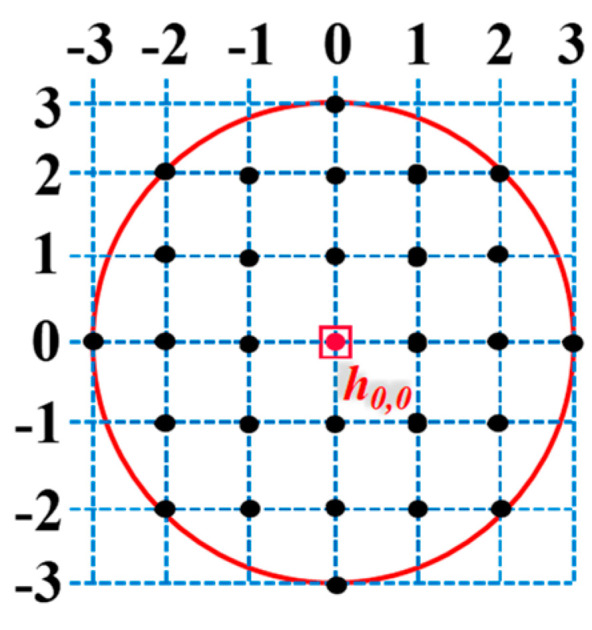
Layout of data acquisition points in the conical defect-opening region.

For the conical defect, the axial signal *B_x_* and radial signal *B*_y_ in the *x*-detection direction are illustrated in Figure 10c,d, respectively. The lift-off values for the multi-sensor probe were selected as follows: *r*_1_ = 1 mm, *r*_2_ = 2 mm, *r*_3_ = 1.5 mm, and *r*_4_ = 2.5 mm. In the defect region depicted in Figure 13, the red line is the profile of the defect opening. there are 7 channels in the *x*-detection direction and 7 channels in the *y*-detection direction, and the total number of data acquisition points *c* is 29. Calculate the defect depth at each acquisition point, identifying the maximum defect depth *h_max_* at point *h_0,0_* (the vertex position of the cone). The calculation results are presented in Table 7.

According to the calculation results in Table 7, under simulated conditions, the MSSF quantification model maintains a fast computation speed, requiring only 2.01 s even when the base of the conical defect is uneven. Furthermore, it exhibits a quantification error of only 6.33% for the maximum defect depth, which meets the requirements for ultra-high-definition MFL detection. This demonstrates that the MSSF quantification model excels in both computation speed and quantification accuracy.

### 4.4. Comparison of the Quantification Accuracy and Efficiency

**Figure 14:**
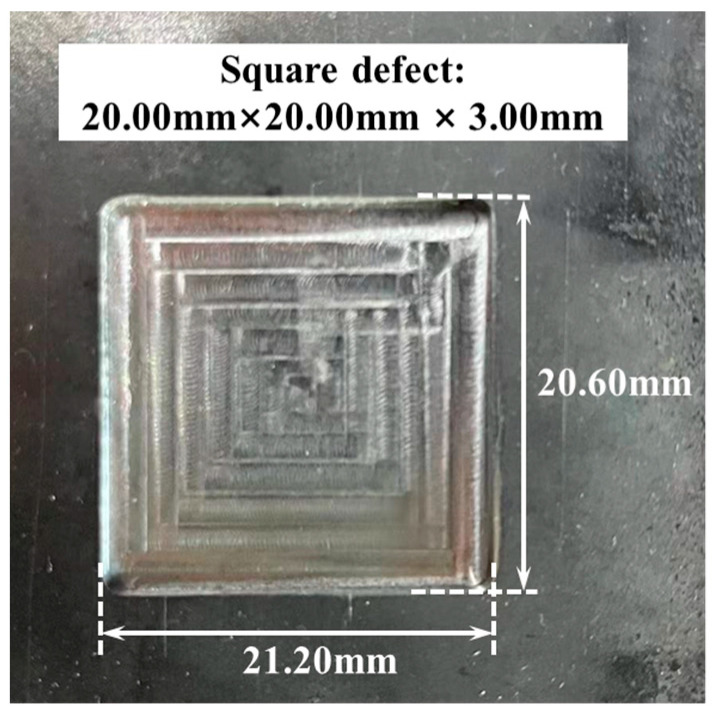
Example of the test defect.

To compare the performance of the MSSF quantification method with other quantification methods, additional comparative experiments are conducted. This study selects the test defect depicted in Figure 14, representing a rectangular defect with dimensions of 2*l* = 21.20 mm, 2*w* = 20.60 mm, and *h* = 3.00 mm. The MFL signals of the test defect are acquired using the MFL detection setup depicted in Figure 11a. Subsequently, the quantification accuracy and iteration speed of the MSSF method were compared with those of the single sensor quantification method, the genetic algorithm (GA) [28], the particle swarm optimization (PSO) [44], and the radial basis function neural network (RBFNN)-based error adjustment (EA) methodology [45]. The specific calculation results are presented in Table 8.

Computer hardware configurations are as follows: CPU-Inter Core i7-12700K CPU @ 3.60 GHz, Memory-32 GB, GPU-NVIDIA GeForce RTX 3060, and hard disk-1 TB. All the computations are implemented in MATLAB R2021(b).

The computational results demonstrate that the quantification accuracies of most methods are satisfactory, except those using single-sensor probes, which exhibit relatively higher quantification errors. Moreover, the computation time of the MSSF method is notably shorter than that of other models. This is attributed to the fact that the MSSF quantification method does not necessitate the iterative optimization of the forward model parameters in the traditional defect inversion method. Instead, it directly incorporates defect information obtained from multi-sensor probes into the derived forward model (MDM), allowing for direct iterative determination of defect size. Furthermore, the solution equation for the MSSF method is simplified, requiring only a single equation to be solved for each element. In a series of experiments, the MSSF quantification method typically required fewer than 15 iterations to calculate the defect depth at each acquisition point. This notably reduces computational complexity, highlighting that the method substantially improves efficiency without compromising accuracy.

### 4.5. Robustness of MSSF Quantification Method

**Figure 15:**
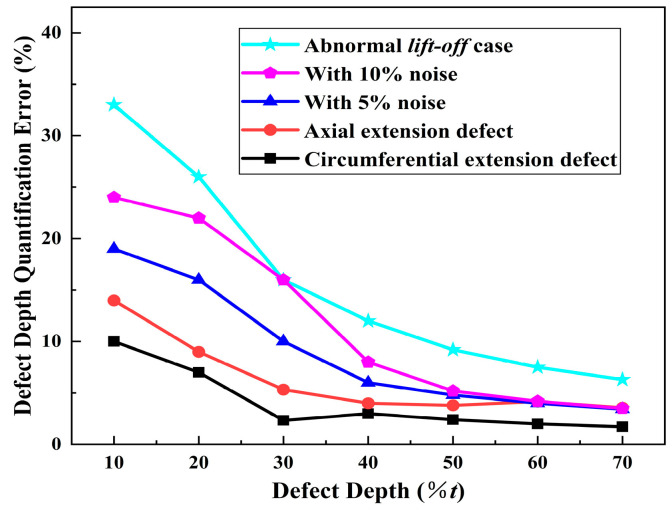
Quantification error of MSSF method for different defect depths.

This section examines the robustness of the MSSF quantification method in various application scenarios. These scenarios include rectangular defects with varying extension directions: axial defect (2*l* = 10 mm, 2*w* = 4 mm) and circumferential rectangular defect (2*l* = 4 mm, 2*w* = 10 mm), anomalous lift-off values including ultra-high lift-off values of *r*_1_ = 3 mm, *r*_2_ = 4 mm, *r*_3_ = 3.5 mm, *r*_4_ = 4.5 mm, and MFL signals with 5% and 10% noise levels. The relative error of the proposed method for defect depth quantification in the above cases was analyzed and calculated. The specimen thickness *t* is 10 mm, and the defect depth ranges from 10%*t* to 70%*t* in steps of 10%*t*. The results are presented in Figure 15.

As illustrated in Figure 15, there is a negative correlation between the relative error and the overall defect depth. For defects with a depth of less than 20%*t*, the amplitude and SNR of the MFL signal are low, resulting in a substantial relative error in quantification. Nevertheless, the quantification error of the defects under different extension directions consistently remains below 15%. When the defect depth exceeds 20%*t*, the quantification error remains within 10%. Regarding conditions involving noise and anomalous lift-off values, the reduction in SNR results in diminished characteristic information such as extreme and inflection points. Consequently, this adversely affects the quantification accuracy. However, when the defect depth is increased to 40%*t*, the relative error of the depth quantification for both 5% and 10% noise remains below 10%. Even under conditions of abnormal lift-off values, the relative error remains below 15%.

Furthermore, the figure illustrates that anomalous lift-off values have the most significant impact on the quantification accuracy. Specifically, when the defect depth is only 10%*t*, the relative error in depth quantification is 33.4%, with an absolute error of 3.3%*t*. Conversely, when the defect depth reaches 70%*t*, the relative error decreases to 6.3%, and the absolute error is 4.4%*t*. These results are within the acceptable range for ultra-high-definition MFL detection. Additionally, the MSSF quantization method demonstrates notable robustness even under complex detection conditions.

## 5. Conclusions

In this article, an MSSF quantification method is proposed to achieve a fast estimation of defect size in MFL detection. The method constructs a multi-sensor probe system that can acquire a variety of lift-off value MFL signals, which increases the diversity of defect information and thus reduces the complex iterative process. Subsequently, a method for iteratively solving the defect dimensions is derived based on a 3D MDM combined with the structural characteristics of the multi-sensor probe. The proposed method is validated by FEM simulations and physical experiments. The results demonstrate that the method accurately and efficiently estimates defect sizes, typically requiring fewer than 15 iterations per depth calculation point within the defective region. Rectangular metal loss, circular perforation, square perforation, and conical metal loss defects are quantified with an error rate of less than 10%. In comparison to other prevalent quantification methodologies, this approach not only guarantees accuracy but also enhances efficiency.

## Figures and Tables

**Table 1:** Material parameters for each component.

Part	Relative Magnetic Permeability	Remanent Magnetization
Magnet	1	1.3 T
Air	1	-
Brush	3000	-
Yoke iron	5000	-
Steel pipe	shown in the *B-H* curve	-

**Table 2:** Quantification results and relative errors for the rectangular metal loss defect using simulation data.

	Defect Dimension (mm)			Computing Time (s)
	*l*	*w*	*h_max_*	
**True values**	2.00	5.00	3.00	4.12
Calculated values	2.08	5.06	2.88	
Relative error	4.00%	1.20%	3.83%	

**Table 3:** Quantification results and relative errors for the circumferential rectangular groove defect using experimental data.

	Defect Dimension (mm)			Computing Time (s)
	*l*	*w*	*h_max_*	
**True values**	3.00	15.00	2.40	16.10
Calculated values	3.26	16.10	2.22	
Relative error	8.67%	7.33%	7.58%	

**Table 4:** Quantification results and relative errors for the axial rectangular groove defect using experimental data.

	Defect Dimension (mm)			Computing Time (s)
	*l*	*w*	*h_max_*	
**True values**	15.00	3.00	2.40	14.31
Calculated values	15.30	3.22	2.30	
Relative error	2.00%	7.33%	4.17%	

**Table 5:** Quantification results and relative errors for the square perforation defect using simulation data.

	Defect Dimension (mm)			Computing Time (s)
	*l*	*w*	*h_max_*	
**True values**	3.00	3.00	3.00	3.76
Calculated values	3.12	3.07	2.89	
Relative error	4.00%	2.33%	3.78%	

**Table 6:** Quantification results and relative errors for the circular perforation defect using experimental data.

	Defect Dimension (mm)		Computing Time (s)
	*R*	*h_max_*	
**True values**	2.50	6.00	1.04
Calculated values	2.27	5.72	
Relative Error	8.80%	4.63%	

**Table 7:** Quantification results and relative errors for the conical defect using simulation data.

	Defect Dimension (mm)		Computing Time (s)
	*R*	*h_max_*	
**True values**	3.00	3.00	2.01
Calculated values	2.96	2.81	
Relative Error	1.33%	6.33%	

**Table 8:** Comparative analysis of the results of different quantification methods.

Quantification Method	Estimated Dimension (mm)						Computing Time (s)
	*l*	Error	*w*	Error	*h*	Error	
**True values**	10.60	-	10.30	-	3.00	-	-
Single-sensor	8.93	15.75%	8.67	15.83%	2.44	18.67%	84
GA	11.32	6.79%	10.50	1.94%	3.30	10.00%	186
PSO	11.50	8.49%	10.00	2.91%	3.20	6.67%	164
RBFNN-EA	9.87	6.89%	10.00	2.91%	3.20	6.67%	97
MSSF	**10.00**	**5.66%**	**9.85**	**4.37%**	**2.91**	**3.00%**	**32**

## Data Availability

Due to the confidentiality requirements of the project responsible unit, the data provided in this study should be provided at the request of the corresponding author, because the project has not yet been concluded.
